# Identification of Ginger (*Zingiber officinale* Roscoe) Reference Genes for Gene Expression Analysis

**DOI:** 10.3389/fgene.2020.586098

**Published:** 2020-11-03

**Authors:** Yao Lv, Yanyan Li, Xiaohui Liu, Kun Xu

**Affiliations:** ^1^College of Horticulture Science and Engineering, Shandong Agricultural University, Tai’an, China; ^2^Collaborative Innovation Center of Fruit & Vegetable Quality and Efficient Production in Shandong, Tai’an, China; ^3^Key Laboratory of Biology of Horticultural Crops in Huanghuai Region, Ministry of Agriculture and Rural Affairs, Tai’an, China; ^4^State Key Laboratory of Crop Biology, Tai’an, China; ^5^School of Environment, Tsinghua University, Qingdao, China

**Keywords:** gene expression, ginger, normalization, qRT-PCR, reference gene

## Abstract

Quantitative real-time PCR (qRT-PCR) is widely used in the detection of gene expression level. However, there is no suitable ginger reference gene for qPCR analysis. Therefore, it is the primary task to select and validate the appropriate ginger reference gene to normalize the expression of target genes. In this study, 14 candidate reference genes were selected and analyzed in different tissues (leaf, and rhizome), different development stages, different varieties, and abiotic stress (ABA and salt stress). Expression stability was calculated using geNorm and NormFinder, Bestkeeper, and RefFinder. For abiotic stress and total conditions, 28S and COX were identified as the most stable genes. In addition, RPII was the most stable in the different development stages and different varieties. TEF2 and RPL2 were the least stably expressed in the tissue and all the conditions. In order to verify the feasibility of these genes as reference genes, we used the most stable and least stable reference genes to normalize the expression levels of *ZoSPS* genes under different conditions. This work can provide theoretical support for future research on ginger gene expression.

## Introduction

Gene expression analysis is an important means to reveal plant signal transduction, metabolic pathways, tissue development, and plant stress response (Crisp et al., 2017; Guo et al., 2017; Zachgo et al., 2018). Quantitative real-time PCR (qRT-PCR) is currently the main method for studying gene expression, because of its high sensitivity, strong specificity, and accurate quantification. However, this technique requires stable expression of endogenous genes as expression correction (Liang et al., 2018; Su et al., 2019). Therefore, choosing a suitable reference gene plays an important role in avoiding errors in experimental operations.

A good reference gene should be stably expressed in tissues and cells, and its expression level is not influenced by internal and external conditions (Kozera and Rapacz, 2013). Ribosomal RNAs, actin, ubiquitin, elongation factor-1α, tubulin, etc., are often used as reference genes. However, the stability of these commonly used reference genes varies in different species and conditions (Tian et al., 2015; Zhang et al., 2015). Moreover, due to the limitation of the genome of non-model plants, the reference genes reported in model plants were often selected (Li H. et al., 2018); however, its stability as a housekeeper gene needs to be further explored. If an inappropriate reference gene was selected, the target gene expression will be misdescribed (Petriccione et al., 2015). Therefore, we should choose a suitable reference gene according to actual conditions.

Ginger is widely cultivated in China. It has important uses in daily diet, industrial product production, traditional Chinese medicine, and so on (Wang et al., 2015; Marx et al., 2017; Shukla et al., 2019). However, there is a lack of research on ginger in the world, mainly due to the limitation of genome and the weak foundation of molecular biology. Fortunately, the development of transcriptome technology has enabled ginger to develop at the gene level. However, there is no report on the selection of ginger reference genes, so it is urgent to find a reference gene suitable for ginger gene expression analysis.

In this study, 14 candidate genes [RNA polymerase-II (RPII), clathrin adaptor complex (CAC), exocyst complex component sec3 (SEC3), tonoplastic intrinsic proteins-41 (TIP41), ADP-ribosylation factor (ARF), ribosomal protein L (RPL2), ubiquitin extension protein (UBI), translation elongation factor 2 (TEF2), cytochrome c oxidase subunit Vc (COX), actin (ACT), 28SrRNA (28S), elongation factor 1 α promoter (EF1α), glyceraldehyde 3-phosphate dehydrogenase (GAPDH), and tubulin α-2 (TUB)] were selected for screening and identification, and their expression stability was tested by qRT-PCR. The tissues (leaf and rhizome) were collected 60, 110, 140, and 180 days after sowing. In addition, abiotic stress (ABA treatment and salt stress) and samples of different varieties were also studied. In this study, the expression stability of candidate genes was calculated using geNorm, NormFinder, and Best Keeper. Finally, in order to validate the reference genes, the most and least stable genes or genome combinations were selected to standardize the expression levels of *ZoSPS* genes in different tissues, stages, varieties, and abiotic stress.

## Materials and Methods

### Plant Material, Culture Conditions, and Sample Collection

*Zingiber officinale Roscoe* cultivar “Shannong 1” (S1), “Laiwu big ginger” (LBG), and “Laiwu small ginger” (LSG) were sown in pots (diameter: 25 cm, height: 30 cm) on May 1, 2019. Tissues (leaf and rhizome) were collected 60, 110, 140, and 180 days after sowing. On August 13, S1 with similar growth was selected for ABA treatment [foliar spraying of ABA (100 μmol L^–1^)] and salt stress [soil applying NaCl (100 mmol L^–1^) to simulate salt stress]. Ginger leaves treated with ABA and salt stress were collected on August 20. All samples were collected, washed, and surface dried, then stored at −80°C. Each sample was repeated three times.

### Total RNA Extraction and cDNA Synthesis

Total RNA was extracted using a RNA Isolation Kit (TianGen, China). A total of 400 ng RNA was reverse transcribed to cDNA using a cDNA synthesis kit (TianGen, China).

### Identification of Candidate Reference Genes

A total 14 candidate reference genes, including RNA polymerase-II (RPII), clathrin adaptor complex (CAC), exocyst complex component sec3 (SEC3), tonoplastic intrinsic proteins-41 (TIP41), ADP-ribosylation factor (ARF), ribosomal protein L (RPL2), ubiquitin extension protein (UBI), translation elongation factor 2 (TEF2), cytochrome c oxidase subunit Vc (COX), actin (ACT), 28SrRNA (28S), elongation factor 1 α promoter (EF1α), glyceraldehyde 3-phosphate dehydrogenase (GAPDH), and tubulin α-2 chain (TUB) were selected based on previous reports in other plant species (Chandna et al., 2012; Park et al., 2012; Xu et al., 2012; Tang et al., 2017). Preliminary sequence information of the 14 selected ginger reference genes was blasted using our unpublished transcriptome data.

### qRT-PCR Primer Design and Validation

Quantitative primers for the 14 genes were designed by the Premier 5.0 program. A standard curve using a series of gradient-diluted cDNAs was generated to calculate the gene-specific PCR amplification efficiency (E) and correlation coefficients (R^2^) for each gene, qPCR was performed using the ABI Q6 Real-Time PCR system and a TB GREEN Premix Ex Taqreal-time PCR Kit (Takara, China), and the presence of a single amplification product of the expected size for each gene was verified by electrophoresis on a 1.5% agarose gel. PCR was performed under the following conditions: 95°C for 30 s, followed by 40 cycles at 95°C for 5 s for denaturation and 60°C for 30 s for annealing and extension. The experiments used independent RNA samples from three biological replicates, and mean Ct values were calculated. The primers are shown in Table 1.

**TABLE 1:** The primer sequences and amplification characteristics of 14 candidate reference genes.

Gene name	Primer sequence (5′→3′)	Gene length (bp)	PCR efficiency (%)	Correlation coefficient (*R*^2^)	Slope (k)
*28S*	FP: 5′CCACTTATCCTACACCTCTC3′	136	98.9	0.999	−3.367
	RP:5′CACTGTCCCTGTCTACTATC3′				
*EF-1*α	FP: 5′GATGGACAGACACGAGAA3′	137	99.23	0.995	−3.259
	RP:5′GAGACCTCCTTGACGATT3′				
*UBI*	FP: 5′GCGGACTACAACATACAGA3′	130	99.18	0.984	−3.386
	RP:5′GCTTGACCTTCTTCTTCTTG3′				
*ARF*	FP: 5′GGCATTACTTCCAGAACAC3′	110	100.12	0.992	−3.355
	RP:5′CTCATCCTCATTAAGCATCC3′				
*RPII*	FP: 5′CTGCTGATGGATACGAATG3′	132	99.35	0.98	−3.469
	RP:5′CTGCCCAAGAGAATGAAAG3′				
*GAPDH*	FP: 5′CATTCCGTGTTCCAACTG3′	150	99.05	0.99	−3.312
	RP:5′CCAAGTCCTCATCCACATA3′				
*CAC*	FP: 5′GAAGTATCGCATAACTGAGG3′	82	97.24	0.997	−3.107
	RP:5′TTCCATCCGTGTTCTACC3′				
*TUB*	FP: 5′CAACCATCAAGACGAAGAG3′	82	98.84	0.981	−3.351
	RP:5′GGTGCCTGATAGTTAATTCC3′				
TEF2	FP: 5′GTTGTCTCCTACCGTGAA3′	133	98.05	0.979	−3.611
	RP:5′CGTTGTCAATGTCCTCAG3′				
ACT	FP: 5′CACTGATTGCCTGATGAAG3′	130	98.76	0.988	−3.411
	RP:5′CTCCAACTCCTGTTCGTA3′				
TIP41	FP: 5′CGCTACTGGCTTAGAGTT3′	90	98.06	0.981	−3.335
	RP:5′CGGCATTTCCTTGTCATC3′				
COX	FP: 5′TTAGACAAGGAGCGAAGG3′	135	101.26	0.993	−3.485
	RP:5′CGAGGATAGTGCATGTAGTA3′				
SEC3	FP: 5′TTAGGAGCCAGAGTGTTG3′	116	98.88	0.99	−3.326
	RP:5′CTTAGGAAGACGCTGTGA3′				
RPL2	FP: 5′GGAGGTCATAAGCGTCTAT3′	144	97.91	0.992	−3.386
	RP:5′ATATCTCTTCTCACCATCCC3′				

### Data Analysis

The Ct values of each reference gene were used to evaluate their expression levels. Expression stability was analyzed using the geNorm (Vandesompele et al., 2002), NormFinder (Andersen et al., 2004), BestKeeper (Pfaffl et al., 2004), and RefFinde^1^.

### Validation of the Candidate Reference Genes

In order to verify the results of our experiments, the most stable and unstable reference genes were selected to validate the expression of the sucrose phosphate synthase (SPS) gene in different tissue samples (leaf, and rhizome), different stages, different varieties, and abiotic stress. The primers for *ZoSPS* were forward 5′ CAGAATGCCAAGGATTAGC3′ and reverse 5′ CCGTATGAGACCGTGAAT3′, and the SPS gene (GeneBank accession no. GI161176315) was blasted using our unpublished transcriptome data (Verma et al., 2011). The qRT-PCR experimental method was the same as described above, and the relative expression level was calculated by 2^–ΔΔct^ method. Data from three biological replicates were analyzed using analysis of variance (ANOVA).

## Results

### Reference Gene Expression Analysis

The target specificity of 14 reference genes was detected by gel electrophoresis (Supplementary Figure S1). There was a single band around 100 bp in 14 genes. In addition, the 14 genes produced a single peak on their respective melting curves, which indicates that the primers are very specific (Supplementary Figure S2).

The amplification efficiency of 14 candidate primer pairs was assessed. Each primer pair was amplified, and cDNA was serially diluted 5 times in 10-fold iterations. The amplification efficiencies of 14 candidate reference genes ranged between 96 and 101%, and the regression coefficients (R^2^) were about 0.99 (Table 1). These results showed that all the 14 candidate primers met the basic requirements of reference genes.

**FIGURE 1:**
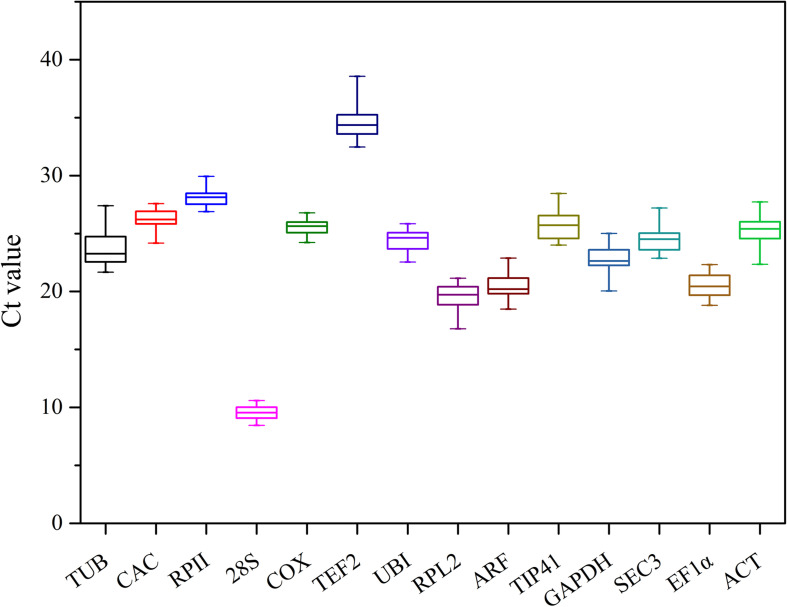
Expression levels of 14 candidate reference genes in ginger. The lines across the box indicate median values; boxes depict 25/75 percentiles. Whisker caps indicate the minimum and maximum values.

The average Ct distributions of 14 genes in ginger samples were plotted (Figure 1). The results indicated that 14 candidate reference genes have average Ct thresholds between 8 and 35. Here, 28S, TUB, RPL2, ARF, GAPDH, SEC3, EF1α, and UBI have average Ct thresholds lower than 25. The gene with the highest average Ct value was TEF2.

### Evaluation of Reference Gene Stability Using Genorm, Bestkeeper, and NormFinder

#### GeNorm Analysis

**FIGURE 2:**
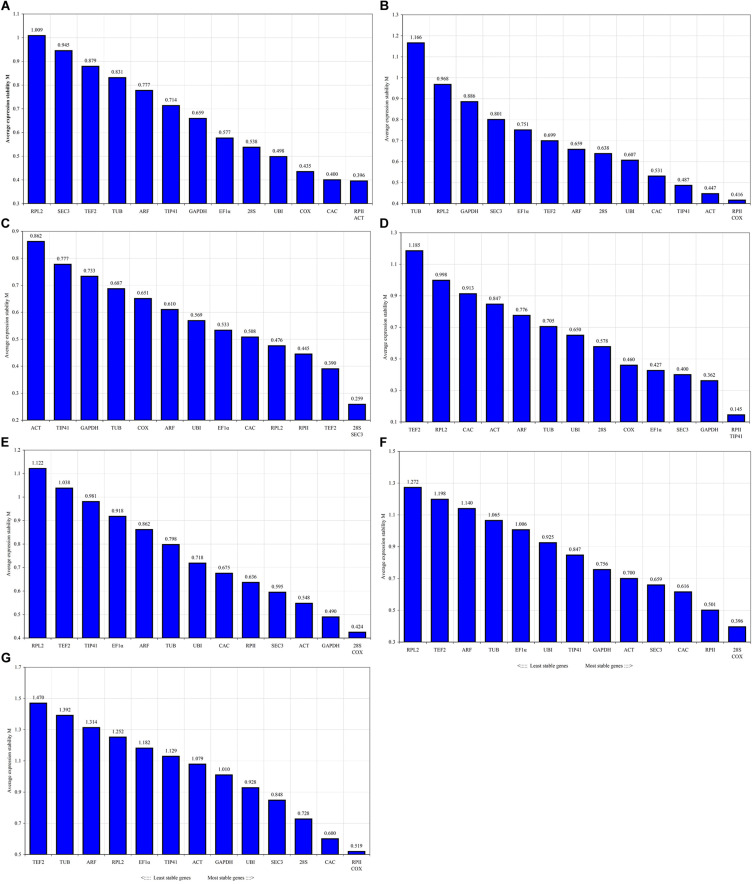
Gene expression stability calculated by geNorm. Mean expression stability (M) was calculated following stepwise exclusion of the least stable gene in different conditions. The least stable genes are on the left and the most stable genes on the right. **(A)** Gene expression stability in rhizome at different developmental stages. **(B)** In leaf at different developmental stages. **(C)** In rhizome of different varieties. **(D)** In leaf of different varieties. **(E)** Under ABA treatment. **(F)** Under salt stress. **(G)** Total conditions.

GeNorm calculates the M value through pairwise comparison in stepwise iterations and screens out genes with better stability (Vandesompele et al., 2002). The lower the M value, the more stable the expression for the gene. The M value of the stable gene should be lower than 1.5 (Vandesompele et al., 2002). This study showed that the M values of all candidate genes under different conditions were lower than 1.5. Under different development stages, ACT and RPII had the lowest M value (0.396) and were the most stable genes in rhizomes. In addition, COX and RPII (0.416) were the most stable genes in leaf; however, the least stable genes were RPL2 and TUB (1.009 and 1.166) in rhizome and leaf, respectively (Figure 2). From the perspective of different ginger varieties, SEC3 and 28S (0.259) were the most stably expressed genes in rhizome, and TIP41 and RPII (0.145) were the most stable genes in leaf, while the least stable genes were ACT and TEF (0.862 and 1.185) in rhizome and leaf, respectively. Under salt stress, COX and 28S (0.396) were the most stably expressed, but RPL2 was the least stable (Figure 2). Under ABA treatment, COX and 28S (0.424) were the most stably expressed genes, while RPL2 was the least stably expressed (Figure 2). Under the total conditions, COX and RPII (0.519) were the most stably expressed genes, whereas TEF2 was the least stable.

The pairwise variation (V) between the normalization factors is used to determine the optimal number of reference genes required. Assuming a cutoff of Vn/n + 1 ≤ 0.15, it was determined that the genes required for normalization in different periods and different varieties were the top two reference genes. Under total conditions, the number of genes increased to four (Supplementary Figure S3).

#### NormFinder Analysis

Like geNorm, the larger value, the lower stability. The most stable genes was RPII (rhizome and leaf) for different growth stages, SEC3 (rhizome) and TIP41 (leaf) for different varieties, 28S for ABA treatment and salt stress, and COX for total conditions. Additionally, the second and third stable genes were CAC, ACT (rhizome) and ACT, TIP41 (leaf) for different stages, RPII, EF1α (rhizome) and RPII, COX (leaf) for different varieties, COX and GAPDH for ABA treatment, COX and RPII for salt stress, and 28S and RPII for total conditions, respectively. TEF2 was the least stable genes for total conditions (Table 2).

**TABLE 2:** Analyses of reference genes evaluated according to NormFinder.

	Stages rhizome		Stages leaf		Varieties rhizome		Varieties leaf		ABA		Salt		Total	
Ranking	Gene	SV	Gene	SV	Gene	SV	Gene	SV	Gene	SV	Gene	SV	Gene	SV
1	RPII	0.281	RPII	0.158	SEC3	0.196	TIP41	0.130	28S	0.186	28S	0.214	COX	0.340
2	CAC	0.296	ACT	0.261	RPII	0.201	RPII	0.203	COX	0.238	COX	0.285	28S	0.394
3	ACT	0.324	TIP41	0.268	EF1α	0.211	COX	0.359	GAPDH	0.316	RPII	0.451	RPII	0.420
4	UBI	0.356	COX	0.281	RPL2	0.243	28S	0.361	RPII	0.318	GAPDH	0.500	UBI	0.470
5	28S	0.374	ARF	0.323	CAC	0.311	UBI	0.409	ACT	0.356	UBI	0.509	CAC	0.542
6	EF1α	0.374	UBI	0.361	28S	0.333	GAPDH	0.413	UBI	0.457	SEC3	0.555	SEC3	0.662
7	COX	0.378	28S	0.419	TEF2	0.348	EF1α	0.433	SEC3	0.477	TIP41	0.578	GAPDH	0.676
8	TIP41	0.495	CAC	0.465	ARF	0.365	TUB	0.470	CAC	0.496	CAC	0.608	ACT	0.701
9	TUB	0.504	TEF2	0.527	UBI	0.405	SEC3	0.481	TUB	0.610	TUB	0.624	EF1α	0.723
10	GAPDH	0.504	EF1α	0.535	COX	0.452	ARF	0.578	ARF	0.627	ARF	0.686	TIP41	0.736
11	TEF2	0.635	SEC3	0.596	TUB	0.515	CAC	0.725	TIP41	0.699	EF1α	0.724	RPL2	0.740
12	ARF	0.645	RPL2	0.793	GAPDH	0.524	ACT	0.817	TEF2	0.713	TEF2	0.730	ARF	0.746
13	RPL2	0.817	GAPDH	0.924	TIP41	0.615	RPL2	0.840	EF1α	0.754	ACT	0.813	TUB	0.915
14	SEC3	0.891	TUB	1.463	ACT	0.752	TEF2	1.227	RPL2	0.851	RPL2	0.890	TEF2	0.946

*SV means stability value.*

#### BestKeeper

BestKeeper estimates the stability of candidate reference genes by standard deviation (SD) and variation coefficient (CV). The lower the SD value and the CV value, the stronger the stability of the reference gene, and the reference gene with an SD value > 1 is often regarded as an unstable expression gene. It was found that ARF and RPII were the most stably expressed genes for the different stages in rhizome, and RPII and TIP41 were the most stably expressed genes for the different stages in leaf. However, SEC3 and TUB were identified as the least stable genes in rhizome and leaf, respectively. Under different varieties, the most stably expressed genes were RPII (rhizome) and TIP41 (leaf), and TEF2 (rhizome) and RPII (leaf) were ranked in the second positions, whereas ACT (rhizome) and TEF2 (leaf) were identified as the least stably expressed genes. In addition, RPII was the most stably expressed under ABA treatment, followed by 28S as the second positions. However, RPL2 with the SD values > 1 was unstably expressed. Under salt stress, 28S was the most stably expressed gene, while RPL2 was treated as the unstably expressed genes. Nevertheless, TEF2 displayed a higher SD value under total conditions. 28S, COX, and RPII owing a lower SD value was considered as the stably expressed genes (Table 3).

**TABLE 3:** The ranking of 14 candidate reference genes at different conditions according to BestKeeper.

		TUB	CAC	RPII	28S	COX	TEF2	UBI	RPL2	ARF	TIP41	GAPDH	SEC3	EF1α	ACT
Stages rhizome	SD [ ± CP]	1.02	0.55	0.53	0.53	0.6	0.75	0.63	0.94	0.45	0.8	0.64	1.35	0.7	0.68
	CV [% CP]	4.42	2.05	1.86	5.61	2.33	2.21	2.6	4.84	2.32	2.99	2.8	5.45	3.56	2.65
	*p*-value	0.001	0.003	0.001	0.009	0.001	0.443	0.001	0.315	0.614	0.003	0.101	0.013	0.05	0.001
	Ranking	13	4	2	3	5	10	6	12	1	11	7	14	9	8
Stages leaf	SD [ ± CP]	1.98	0.46	0.18	0.45	0.37	0.63	0.4	1	0.4	0.34	1.17	0.78	0.74	0.37
	CV [% CP]	8.01	1.72	0.62	4.77	1.45	1.82	1.71	5.57	1.97	1.38	5.18	3.11	3.48	1.46
	*p*-value	0.529	0.781	0.011	0.679	0.027	0.463	0.537	0.442	0.727	0.358	0.38	0.24	0.139	0.06
	Ranking	14	8	1	7	3	9	5	12	6	2	13	11	10	4
Varieties rhizome	SD [ ± CP]	0.41	0.29	0.25	0.44	0.59	0.28	0.72	0.49	0.6	0.72	0.55	0.33	0.4	1.25
	CV [% CP]	1.8	1.12	0.88	4.82	2.33	0.8	3	2.46	3.04	2.7	2.47	1.41	2.05	4.82
	*p*-value	0.317	0.568	0.157	0.014	0.137	0.644	0.005	0.001	0.084	0.939	0.898	0.006	0.015	0.001
	Ranking	6	3	1	7	10	2	12	8	11	13	9	4	5	14
Varieties leaf	SD [ ± CP]	0.51	0.91	0.17	0.42	0.39	1.78	0.52	1.14	0.84	0.12	0.51	0.49	0.39	0.86
	CV [% CP]	2.18	3.55	0.61	4.31	1.55	5.06	2.14	6.1	3.93	0.48	2.25	2.03	1.84	3.55
	*p*-value	0.015	0.729	0.13	0.178	0.818	0.003	0.048	0.026	0.027	0.879	0.057	0.002	0.239	0.002
	Ranking	7	12	2	5	3	14	9	13	10	1	8	6	4	11
ABA	SD [ ± CP]	0.88	0.41	0.26	0.4	0.43	0.63	0.92	1.31	1.07	0.73	0.53	0.69	1.08	0.61
	CV [% CP]	3.8	1.55	0.94	4.29	1.7	1.84	3.77	6.79	5.19	2.84	2.25	2.84	5.31	2.39
	*p*-value	0.007	0.616	0.26	0.001	0.003	0.007	0.001	0.055	0.001	0.697	0.002	0.07	0.16	0.002
	Ranking	10	3	1	2	4	7	11	14	12	9	5	8	13	6
Salt	SD [ ± CP]	0.66	0.59	0.28	0.24	0.46	1.26	0.98	1.33	1.04	0.72	0.77	0.85	0.95	0.67
	CV [% CP]	2.89	2.26	1	2.65	1.84	3.51	4.01	6.87	5.05	2.78	3.28	3.5	4.63	2.76
	*p*-value	0.495	0.925	0.122	0.004	0.01	0.005	0.001	0.049	0.143	0.587	0.026	0.082	0.143	0.138
	Ranking	5	4	2	1	3	13	11	14	12	7	8	9	10	6
Total	SD [ ± CP]	1.22	0.62	0.51	0.45	0.47	1.24	0.7	1.03	0.93	1.02	0.93	0.88	0.87	0.97
	CV [% CP]	5.15	2.38	1.82	4.71	1.84	3.56	2.87	5.3	4.53	3.94	4.04	3.59	4.23	3.84
	*p*-value	0.084	0.24	0.06	0.001	0.001	0.46	0.001	0.002	0.167	0.017	0.001	0.002	0.14	0.054
	Ranking	13	4	3	1	2	14	5	12	8	11	9	7	6	10

*SD means standard deviation, CV means coefficient of variance.*

#### Comprehensive Analysis

We use RefFinder to determine the comprehensive ranking of candidate reference genes (Table 4). RefFinder is an online software, which combines the algorithms of GeNorm, Bestkeeper, NormFinder, and other software. RefFinder ranks each individual gene in a variety of ways to calculate the stability value of the reference gene, and the smaller the stability value, the more stable the gene. RPII was identified as the most stably expressed for different stages and different varieties. However, 28S and COX were identified as the most stably expressed genes under ABA treatment and salt stress and for total samples. Nevertheless, TEF2 and RPL2 were the most unstably expressed genes in ginger.

**TABLE 4:** The ranking of 14 candidate reference genes at different conditions using RefFinder software.

	Stages rhizome		Stages leaf		Varieties rhizome		Varieties leaf		ABA		Salt		Total	
Ranking	Gene	GRV	Gene	GRV	Gene	GRV	Gene	GRV	Gene	GRV	Gene	GRV	Gene	GRV
1	RPII	1.190	RPII	1.000	SEC3	1.410	TIP41	1.000	28S	1.190	28S	1.000	COX	1.190
2	CAC	2.630	COX	2.380	RPII	2.000	RPII	1.680	COX	2.000	COX	1.860	28S	2.000
3	ACT	3.560	TIP41	2.830	28S	3.440	COX	3.570	RPII	3.130	RPII	2.710	RPII	2.280
4	COX	3.940	ACT	3.000	TEF2	4.140	28S	4.860	GAPDH	3.410	SEC3	5.480	CAC	4.160
5	UBI	4.610	ARF	5.890	RPL2	4.360	EF1α	4.950	ACT	4.950	CAC	5.630	UBI	4.680
6	28S	5.050	CAC	5.890	CAC	4.820	GAPDH	5.630	CAC	6.050	UBI	6.670	SEC3	5.960
7	ARF	5.900	UBI	6.480	EF1α	5.380	SEC3	6.450	SEC3	6.650	GAPDH	6.700	GAPDH	7.480
8	EF1α	7.450	28S	7.480	UBI	8.850	UBI	7.090	UBI	7.500	TUB	7.670	EF1α	7.610
9	GAPDH	8.210	TEF2	9.000	TUB	9.450	TUB	8.210	TUB	9.240	TIP41	7.740	ACT	9.460
10	TIP41	8.920	EF1α	10.000	ARF	9.720	ARF	10.000	TIP41	10.190	ACT	7.930	TIP41	9.460
11	TUB	10.940	SEC3	11.000	COX	9.740	ACT	11.490	ARF	10.980	EF1α	10.240	ARF	11.170
12	TEF2	11.470	RPL2	12.240	GAPDH	11.170	CAC	11.490	TEF2	11.140	ARF	12.000	RPL2	11.240
13	RPL2	13.220	GAPDH	12.740	TIP41	13.000	RPL2	13.000	EF1α	11.980	TEF2	13.000	TUB	13.000
14	SEC3	13.490	TUB	14.000	ACT	14.000	TEF2	14.000	RPL2	14.000	RPL2	14.000	TEF2	14.000

*GRV means geomean of ranking value.*

### Validation of Candidate Reference Genes

**FIGURE 3:**
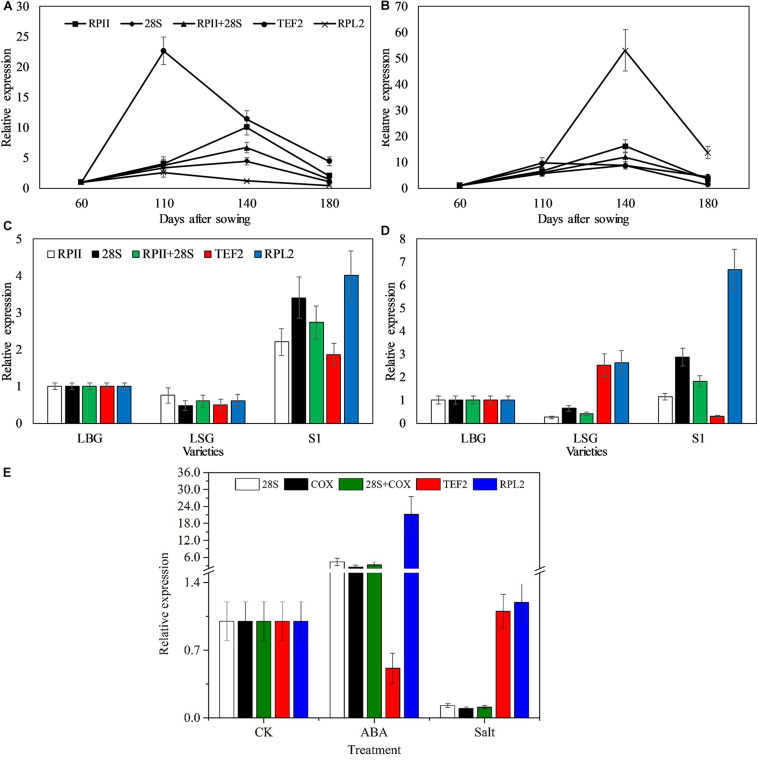
Relative expression of *ZoSPS* in different conditions. RPII, 28S, and COX were used as one or two most stable reference genes; TEF2 and RPL2 were used as the least stable reference gene. **(A)** In rhizome at different developmental stages. **(B)** In leaf at different developmental stages. **(C)** In rhizome of different varieties. **(D)** In leafs of different varieties. **(E)** ABA and salt stress treatment.

In order to verify the reliability of the reference genes selected in this study, we selected the SPS gene for verification. As is well known, SPS is the key control gene in the synthesis of sucrose (Sawitri et al., 2018). It plays an important role in sink-source metabolism and sucrose accumulation (Li X. et al., 2018; Verma et al., 2019), and its expression and gene-coding enzyme activity were significantly different in different tissues and at different stages (Laporte et al., 2001; Lester et al., 2001). Therefore, we chose the SPS gene as the target gene to verify the feasibility of the reference gene identified in this study. Under different developmental stages, the expression of *ZoSPS* first increased and then decreased when normalized with the two stable genes (RPII and 28S), and the highest expression was 140 days after sowing. However, the expression pattern changed significantly when normalized with the least stable genes (RPL2 and TEF2) (Figure 3). Under different varieties, the expression of *ZoSPS* was S1 > LBG > LXG when the most stable reference genes (RPII and 28S) were used to normalize, whereas the expression of SPS in LXG leaves was significantly increased using the least stable gene (TEF2 and RPL2) (Figure 3). Through normalization detection of reference genes under ABA treatment and salt stress, we found that both 28S and COX could be used as reference genes for normalization, but the expression of SPS normalized by TEF2 was significantly decreased compared to 28S and COX under ABA treatment (Figure 3). In addition, the relative expression of SPS normalized by TEF2 and RPL2 was increased compared to 28S and COX under salt stress (Figure 3). Our previous studies showed that the SPS activity increased first and then decreased with the growth of ginger, which is consistent with the relative expression of *ZoSPS* normalized by the most stable combination (RPII and 28S) at different stages in this study, indicating the reliability of using RPII and 28S as housekeeping genes.

## Discussion

Due to the lack of molecular biology research in ginger, there is no suitable reference gene for PCR analysis. This is the first time to screen and identify a series of candidate genes through stability analysis to determine the best reference gene for ginger. In addition, there are many problems in the process of ginger cultivation, such as long cultivation cycle, numerous varieties, and salt stress. Quantitative detection of the expression of key genes by qRT-PCR can solve the response mechanism of ginger under different conditions. The optimal reference gene can ensure the accurate presentation of gene expression in qRT-PCR analysis (Wang et al., 2020).

Appropriate reference genes contribute to more accurate expression of target genes (Connors et al., 2019). The stability of commonly used reference genes in different plants or different environments is different. For example, GAPDH of *Cyamopsis tetragonoloba* can be stably expressed under high temperature and salt stress (Jaiswal et al., 2018), but the stability of GAPDH of *Setaria viridis* was the lowest (Martins et al., 2016).

We used the geNorm, NormFinder, and BestKeeper programs to analyze the stability of candidate reference genes. However, there were some differences in the stability rankings of the reference genes given by the three softwares, mainly because of the different statistical algorithms. These analysis differences also appeared in other studies (Li et al., 2016; Hu et al., 2018). Through comprehensive analysis, the most stable RPII, 28S, and COX and the least stable RPL2 and TEF2 were selected for subsequent verification.

Commonly used reference genes (actin, tubulin, ubiquitin, etc.) have always been very popular. However, these genes have defects, and their expression varies greatly in different tissues, different developmental stages, and different experimental conditions (Chapman and Waldenström, 2015; Li et al., 2019). This is mainly related to the variability of reference genes in different environments and species. In addition, in the studies of reference gene stability, these commonly used reference genes are often not the most optimal choice (Chapman and Waldenström, 2015; Adeyinka et al., 2019; Gao et al., 2020), and this study has the same findings. In the qRT-PCR research on ginger, common reference genes (TUB, actin, EF1α, etc.) have been used (Girija et al., 2017; Jiang et al., 2017; Chen et al., 2020), but this study found that these are not the best choice, and some common reference genes even rank lower in this study. Therefore, the selection of appropriate reference genes in ginger is very important for the standardization of target genes.

This study found that the RPII gene ranked first or second by the RefFinder program was proved to be the best reference gene for different stages, different varieties, ABA treatment, and salt stress. In addition, 28S also normalized the expression of target genes. RPII is the enzyme that transcribes mRNA from protein-encoding genes (Adelman and Lis, 2012), which is the main reason why they are continuously expressed and show minimal changes. Xu et al. (2012) found that RPII is a relatively stable gene in radish. Therefore, RPII can be stably expressed in different periods and different varieties of ginger. 28SrRNA is a ribosomal RNA with weak mutation and could not highly modify the total RNA level (Thellin et al., 1999). The expression level of 28SrRNA was very stable compared with the commonly used reference genes (Stürzenbaum and Kille, 2001), so it was often used as a reference gene for quantitative research (González et al., 2002; Klok et al., 2002). This study found that 28S can also be stably expressed under different conditions. There are few studies about COX. Park et al. (2012) found that it can be stably expressed in sweet potato, which is consistent with the results of ABA treatment and salt stress in this study.

TEF2 is a regulatory protein of the translation extension step (Browne and Proud, 2002), which catalyzes the movement of ribosomes along mRNA. However, TEF2 is regulated by a variety of mechanisms (Yin et al., 2003; Jørgensen et al., 2006) and is sensitive to oxidative stress (Parrado et al., 1999). TEF2 promotes protein synthesis in the stress response and helps cells reduce the adverse effects of oxidative stress (Argüelles et al., 2013). Although Xu et al. (2012) found that it can be stably expressed in sweet potato, this study found that TEF2 would break the normal expression of ginger target genes. RPL is the largest subunit in the ribosome, the lack of ribosomal protein will affect the function of the ribosome to a certain extent, and the loss of important ribosomal protein will cause the disorder of intracellular life activities, the abnormality of the organ, and even the death of the organism. Many studies found that RPLs also showed high expression stability in different stages and abiotic stress (Park et al., 2012; Fu et al., 2013; An et al., 2016). However, in this study, RPL2 is the least stable gene, indicating that the expression stability of common reference genes is significantly different in different crops and under different conditions, which proves the importance of selecting ginger stable housekeeping genes.

## Conclusion

This study reports the selection and verification of reference genes for qPCR in ginger under different tissues, different varieties, and abiotic stress. The stability values of 14 candidate reference genes were analyzed by geNorm, NormFinder, Bestkeeper, and RefFinder. Different programs give slightly different gene stability rankings, but in general, RPII and 28S are the most stable reference genes in different stages and different varieties, and 28S and COX are the most stable reference genes in abiotic stress and total conditions, whereas RPL2 and TEF2 are the least stable. In addition, the reliability of the identified reference genes was verified through determining the expression pattern of *ZoSPS.* Moreover, this study can provide theoretical support for future research on ginger gene expression.

## Data Availability Statement

All datasets presented in this study are included in the article/Supplementary Material.

## Author Contributions

YLv, XL, and KX designed the experiments and wrote the manuscript. YLv and KX selected the material. YLv and YLi performed the experiments. YLv and XL analyzed the data. All authors read and approved the final manuscript.

## Conflict of Interest

The authors declare that the research was conducted in the absence of any commercial or financial relationships that could be construed as a potential conflict of interest.

## Footnotes

1 https://www.leonxie.com/referencegene.php
