# Supplementary material for: Identification of Ginger (Zingiber officinale Roscoe) Reference Genes for Gene Expression Analysis
Source: Front Genet. 2020 Nov 3;11:586098. doi: 10.3389/fgene.2020.586098 (PMC7670040; doi:10.3389/fgene.2020.586098)
Supplement: Supplementary file 1 [file Data_Sheet_1.docx]

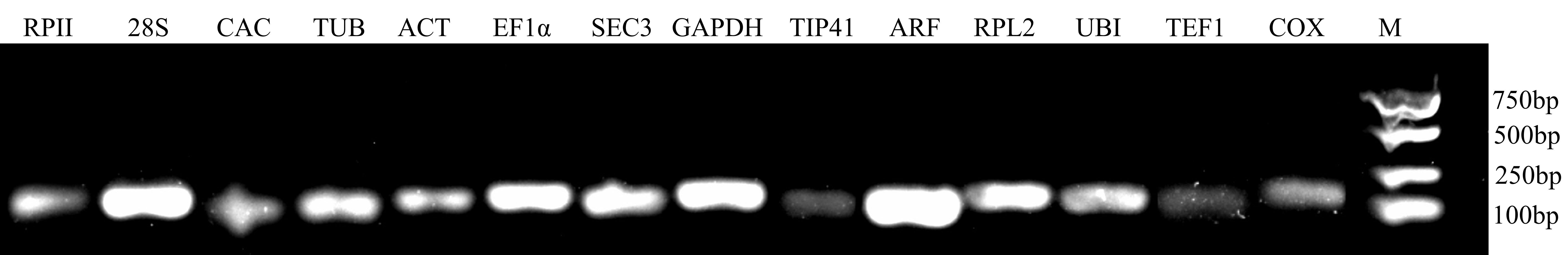


Figure S1 The 14 reference genes were tested for amplicon size using gel electrophoresis.

Figure S2 The 14 reference genes were tested for specificity using melt curve

Figure S3 GeNorm determination of the optimal number of reference genes for qRT-PCR data normalization. Pairwise variation (V) between the normalization factors NFn and NFn+1 was calculated by the geNorm.
